# On the Influence of Extreme Cold on Nervous Functions

**Published:** 1867-09

**Authors:** 


### ARTICLE IV.

### *On the influence of Extreme Cold on Nervous Functions.*

Dr. B. W. Richardson, Senior Physician to the Royal Infirmary for diseases of the chest, has been delivering a series of lectures on this subject, and we present to our readers an abstract of the more important portions of his interesting and valuable remarks.

When any portion of the human body is exposed to cold, it is necessary to reduce its temperature to 16° of Fahrenheit's scale, before actual congelation occurs. By exercising great care, this reduction may be carried to 10° or even 8°, but in every case a rise to 16° occurs at the moment of freezing. In such experiments, the phenomena vary, according to the progress of the reduction of the temperature, and rapidity of the recovery.

He exhibited an experiment on his own arm with two ethers of different rates of volatility. With that which evaporated most slowly, the first phenomenon was a sense of coldness, due to the abstraction of heat from the nervous network. This is followed in a short time by redness and a sense of heat. The plasticity of the blood is diminished, the tension derived from the nerves and exerted on the vessels is reduced; in the part there is increased vascularity, and around the part there is resistance which gives pain. With the ether of rapid evaporation, there is in the part to which it is applied, sudden blanching of the tissue and total insensibility, and in the part surrounding it, redness, exalted sensibility and a slight sensation of burning. In the blanched part the nervous filaments are compressed by the solidification, and the equally compressed capillaries are bloodless. In the reddened part, there is no compression, but the vessels are surcharged with blood, and the sensation is that of a burn or of acute inflammation. On removal of the spray, the white part becomes vascular, it experiences a sensation of heat; and slowly returns to its natural condition.

The duration of these phenomena varies with the subject. In birds, with a higher temperature than that natural to man, the progress of freezing is much slower, while in cold blooded animals, like frogs, the freezing is almost instantaneous. So in men of high, vigorous health, the surface long resists freezing, and in cases of inflammation, like whitlow, it is extremely difficult to congeal the part. In both these cases the reaction is prompt and decided.

In debilitated persons, on the contrary, and especially in the very old, freezing is extremely rapid. The lecturer mentioned one instance in which a gum was frozen in two seconds. Resistance also differs in different parts of the body, and is greatest in those naturally subjected to motion. It is greater in the hands than the arms, in the limbs than in the back.

As for the vascular line surrounding the blanched part, and manifesting increased heat and exalted sensibility, it is in the same condition as the frozen part before congelation, the stage of preaction, as the lecture calls it, or during reaction. At the moment of freezing, the liquid blood of the frozen part is forced out into the surrounding vessels, themselves weakened by the proximity of the cold. Hence arise in these latter vessels, congestion, resistance, friction, and consequently increased developement of heat.

The lecturer tabulates these phenomena as follows:

*1st Stage or Starting point.*

Natural condition:—
Temperature 96° Fahr.
Sensibility perfect.

*2nd Stage, (Preaction.)*

Innervation, removal of nerve-force.
Increased vascularity.
Increased temperature.
Exalted sensibility.

*3rd Stage.*

Quiescence.
No vascularity. No nerve force; no blood.
Temperature 16° Fahr.
Perfect insensibility.
Solidification of water of tissues.

*4th Stage, (Reaction.)*

Returning vascularity of paralyzed vessels.
Increased vascularity.

Increased temperature.
Exalted sensibility.
Re-solution of water of tissues.
Innervation continued.

*5th Stage.*

Return to natural state.
Nervation of vessels.
Reduction of vascularity.
Temperature 96° Fahr.
Natural Sensibility.

When the nervous centres are acted upon by the ether spray, different results are, as might be expected, obtained, as one or another centre is frozen.

Thus when the cerebrum is congealed, the excitability of the spinal cord is greatly augmented. The animal lies on its side, breathing heavily like an apoplectic patient, totally devoid of consciousness, and when undisturbed entirely quiescent. The slightest excitement of the surface however, even a current of air blowing on the body, brought on convulsions, unconscious, painless, co-ordinate, showing the continued influence of the cerebellum, and confined to the voluntary muscles, thus establishing the spinal nature of the excitation.

Majendie, it will be remembered, established the fact, that, when the *corpora striata* were removed, there were violent forward movements of the body. He accounted for this phenomenon by supposing that all forward movements originated in the *cerebellum* or *medulla oblongata* while the backward motions were under the control of the *corpora striata.* Of course the suspension of the influence of these latter bodies, allowed the former to perform their peculiar function unchecked.

Deep freezing of the cerebrum, so as to involve these corpora striata, brought about the forward movements. Sometimes they were slight, resembling the nodding forward of a person asleep in a chair, sometimes they were rapid. When one half of the cerebrum was frozen, irritability of that side of the body was destroyed, while the other side still retained a little.

As far as the cerebrum itself is concerned, the animal is in a sort of artificial hybernation from which it may recover. On recovering, the brain appears to have experienced no injury; the phenomena being simply those of awakening from a profound sleep.

When the cerebellum is frozen, in a pigeon, some stupor occurs, followed by flapping of the wings and backward movements, occurring in distinct paroxysms. In rabbits there are stupor and convulsions, but no backward movements, perhaps because the cerebellum in them is not so easily isolated, and the action of the cold may extend to the corpora striata. These movements depend upon the suspension of the action of the cerebellum which regulates the forward movements, leaving the corpora striata unimpeded in their action on the backward motions. It is really, therefore, the action of the great anterior cerebral ganglia which we see in these cases.

A corroboration of this is to be found in the fact, that, during the stage of preaction, or excitement preceding actual congelation of the cerebellum, forward movements are observed, as they also are during the stage of reaction. The excitability of the cerebellum, under these circumstances, is greater than that of the corpora striata, and therefore, it manifests its own peculiar activity.

When the medulla oblongata is frozen the phenomena are those of disturbed respiration and symptoms of apnœa. The author has never seen a pigeon recover after freezing the medalla oblongata, though Mitchel has. In rabbits, when the cerebellum is subjected to cold, there is first a period of excitement in which the muscles of respiration share. The breathing is first rapid, then the respiratory muscles are contracted, then paralyzed, and the creature dies. Sometime death occurs instantaneously as when the cerebrum and cerebellum are first frozen; a single jet of spray on the medella oblongata will then kill.

The condition of the lungs varies according to circumstances. If the freezing has been gradual they are intensely congested; if rapid, they are collapsed, bloodless and perfectly white.

When the spinal cord is frozen in frogs, stupor and paralysis occur, which gradually disappear upon recovery. In birds, backward movements and stupor occur when the cervical region is frozen. Below the fourteenth vertebra, however, counting from the head, only weakness or tetanic rigidity of the limbs occurs.

When a nerve-cord is frozen gradually, the first phenomnon is overaction, causing sensation at the distal extremity but there is no convulsion. When freezing is complete, sensation is entirely abolished. and the nerve may be cut through, without exciting pain. The frozen nerve is at first slightly coloured, then suddenly becomes intensely white and very hard. Upon thawing, the nerve gradually resumes its natural appearance, without much reaction.

The condition of the nerve materially affects its power to conduct electricity. In the fresh state, as is well known, it is quite a good conductor. When dried, it loses this power completely. When frozen, however, it still conducts electricity, although it cannot convey sensation. If, however, the freezing be continued till the whole calibre of the cord is reduced to a mass of ice, the nerve no longer conducts electricity. In a living animal this gradual change is well-marked. At first the electric current passes freely, decidedly deflects the galvanometer, and produces writhing of the muscles. As the cold increases, the deflection of the needle of the galvanometer diminishes, and so does the twitching of the muscles. At last, when the freezing is complete, the needle is no longer deflected and the muscles cease to twitch. After a time, if the action of the cold be suspended, the parts retnrn to their natural conditions.

One remarkable phenomenon, Dr. Richardson has observed. If the nerve cord be frozen above the part through which the electric current is made to pass, that is to say, nearer to the brain, the motions of the muscles are suppressed, just as if the frozen part itself had formed part of the circuit, and they are restored again upon the return of the congealed portion to its ordinary temperature.

So far we have spoken of the action of cold on compound nerves. In nerves of motion, there is first violent action of the muscles to which they are distributed, then sudden cessation of motion and rigidity until natural temperature is restored.

(To be Continued.)

---

